# Progress in Animal Models of Pancreatic Ductal Adenocarcinoma

**DOI:** 10.7150/jca.37529

**Published:** 2020-01-14

**Authors:** Kaiwen Kong, Meng Guo, Yanfang Liu, Jianming Zheng

**Affiliations:** 1 Pathology Department of Changhai Hospital, Second Military Medical University; 2 Institute of Organ Transplantation, Changzheng Hospital, Second Military Medical University, Shanghai, China; National Key Laboratory of Medical Immunology &Institute of Immunology, Second Military Medical University; 3 Pathology Department of Changhai Hospital, Second Military Medical University; National Key Laboratory of Medical Immunology &Institute of Immunology, Second Military Medical University

## Abstract

As a common gastrointestinal tumor, the incidence of pancreatic cancer has been increasing in recent years. The disease shows multi-gene, multi-step complex evolution from occurrence to dissemination. Furthermore, pancreatic cancer has an insidious onset and an extremely poor prognosis, so it is difficult to obtain cinical specimens at different stages of the disease, and it is, therefore, difficult to observe tumorigenesis and tumor development in patients with pancreatic cancer. At present, no standard protocols stipulate clinical treatment of pancreatic cancer, and the benefit rate of new targeted therapies is low. For this reason, a well-established preclinical model of pancreatic cancer must be established to allow further exploration of the occurrence, development, invasion, and metastasis mechanism of pancreatic cancer, as well as to facilitate research into new therapeutic targets. A large number of animal models of pancreatic cancer are currently available, including a cancer cell line-based xenograft, a patient-derived xenograft, several mouse models (including transgenic mice), and organoid models. These models have their own characteristics, but they still cannot perfectly predict the clinical outcome of the new treatment. In this paper, we present the distinctive features of the currently popular pancreatic cancer models, and discuss their preparation methods, clinical relations, scientific purposes and limitations.

## Introduction

According to NIH statistics, the 5-year survival rate of patients with pancreatic cancer between 2009 and 2015 was only 9.3% in US (https://seer.cancer.gov/statfacts/html/pancreas.html). As such, pancreatic cancer is associated with the worst prognosis of any malignancy because it has an insidious onset, high malignancy, special anatomical location, low resection rate, and high recurrence rate, as well as lack typical symptoms. Furthermore, the incidence of the disease increases annually: by 2030, patients with pancreatic cancer are expected to outnumber those with breast and colorectal cancer in United States, and pancreatic cancer is projected to become the second most common cancer worldwide 1.

Owing to the characteristics of pancreatic cancer, it is difficult for clincians to obtain samples at different stages and to continuously observe the occurrence and development of pancreatic cancer in individual patients. For this reason, animal models of pancreatic cancer help clinicians to further understand the occurrence, development, invasion, and metastasis mechanisms of this disease 1, and can even be used to explore new therapeutic means.

In 1941, Wilson discovered that a diet supplemented with 2-acetylaminofluorene induced pancreatic cancer in albino rats 2. By the late 20^th^ century, as the incidence of pancreatic cancer increased, the study of animal models began to develop, with the help from government agencies.

An ideal animal model of pancreatic cancer should have the following characteristics: (1)Abiological development process similar to that of human pancreatic cancer, which is stable and repeatable. Specifically, pancreatic ductal adenocarcinoma (PDAC) mostly develops from precursor lesions, the most common type being ductal intraepithelial neoplasia (PanINs) 3. Genetic mutations highly correlated with this process have been reported in the literatures 4. At present, a series of mouse pancreatic cancer models have been constructed using genetic engineering technology. By mutating *Kras, Ckn2a, Tp53, Smad4,* and other genes, researchers can induce ductal intraepithelial neoplasia, and the number of mutant genes is highly correlated to the severity of disease 5; (2) Malignant phenotype similar to human tumors, such as anti-apoptotic effect, immune escape, invasion and metastasis. A wide variety of pancreatic cancer cell lines are available on the market, with the phenotype and genotype of each representing a specific subtype of pancreatic cancer. Researchers can infer the mechanism of tumorigenesis and development by studying the relationship between the expression of different specific proteins in cell lines and tumor growth, invasion and metastasis; (3) An experimental method that is easy to implement and efficient in terms of labor and time, as well as a short model establishment period. In particular, pancreatic cancer models used in clinical studies of individualized treatment must have a high success rate and be suitable for large-scale preparation to ensure that they provide evidence regarding individualized treatment options for patients with a short survival time.

## Spontaneous tumor animal models

As used herein, the term “spontaneous tumor” refers to a specific tumor induced spontaneously in a laboratory animal using a chemical, viral induction, or experimental genetic techniques. This contrasts with a transplanted tomor. Spontaneous tumors are more similar to human tumors, so results from animal models of such tumors can be more easily extrapolated to humans. However, the occurrence of spontaneous tumors may vary, so it is difficult to obtain a large amount of tumor material in a short period of time. Moreover, the observation time is long, and the experiment is expensive.

### 1. Chemically induced animal models

**Rat:** Wistar and Lewis rats are injected intraperitoneally with azaserine to induce acinar cell carcinoma of the pancreas, with liver, lung and lymph node metastasis 6, 7. However, the lesions in this model lack a typical duct-like structure and of ten occur alongside tumors of other organs (mammary, liver, kidney). The chemicals 4-hydroxyaminoquinoline-1-oxide 8, nafenopin 9, clofibrate 10, N -(N-methyl-N-nitrosamide)-L-ornithine 11 and different N-nitro compounds 7 can induce acinar cell lesions without a duct-like structure. Vesselinovitch et al. found that topical benzopyrene can induce adenocarcinoma in rats. They implanted dimethylbenzanthracene crystal powder into the pancreas of Sprague-Dawley rats, and approximately 80% of them developed spindle cell sarcoma and poorly differentiated adenocarcinoma. Other researchers using this method have found ductal cell proliferation, tubular adenocarcinoma, acinic cell carcinoma, fibrosarcoma, and invasive ductal adenocarcinoma.

**Hamster:** Hamsters are one of the best animal models for inducing pancreatic cancer. For instance, some carcinogens that work in hamsters are ineffective in other animals, such as rats, mice, Dutch pigs, and rabbits. N-Nitroso-bis(2-oxopropyl)amine(BOP) has the highest specificity in this regard 12, 13, and it show a specific affinity for the pancreas, although its mechanism has not yet been confirmed. This N-Nitroso-BOP model shows unique characteristics that are similar to a well-characterized series of morphologic changes that occurs in the human pancreatic duct, and it frequently shows point mutations in codon 12 of the Kras gene, concurring with findings in human pancreatic cancer 14, 15. Meijers found that the early pseudoductular lesions, induced by BOP in the exocrine pancreas of hamsters originate from proliferating ductal/ductular acinar cells rather than proliferating dedifferentiated acinar cells 16. In addition, the tumors induced in hamsters are most similar to human tumors in terms of morphology, clinical features, and biological manifestations. Not only benign and malignant tumors but also some rare lesions occurred in hamsters. Tumors in hamsters, just as in humans, may show perineural invasion, involvement of the lymph nodes adjacent to the pancreas, weight loss, diarrhea, ascites, and thrombosis. Occasionally, the tumors also involve jaundice, because they mainly occurr in the body and tail of the pancreas. Similar to human tumors, serum antigens CA125, 17-1A, TAG-72, TFGR-α, EGFR, and lectin have been detected in hamster pancreatic tumors, and glucose tolerance has been observed. However, carcinoembryonic antigen, pancreatic cancer embryonal antigen, and α-fetal protein are low or unexpressed 17. Animal models like the hamster model of pancreatic cancer can help identify known and emerging human risk factors and implement appropriate interventions.

### 2. Genetically engineered mouse model of pancreatic cancer

Many recent studies have used genetic technology to introduce oncogenes into mouse embryonic or somatic cells through tissue-specific promoters targeting the pancreas and inducing pancreatic cancer. Genetically Engineered Mouse Models(GEMMs) are constructed using transgenic, gene knock-in, and gene knock-out techniques to transfer specific genes into mice via retroviruses. Most currently used GEMMs are developed using* Kras* proto-oncogenes. The transgenic mice that overexpress the mutant *Kras* gene can mimic pancreatic tumorigenesis. They found that physiological levels of *Kras^G12D^* induce ductal lesions that recapitulate the full spectrum of human pancreatic intraepithelial neoplasias (PanINs), putative precursors to invasive pancreatic cancer 18. As most human pancreatic cancers are ductal adenocarcinomas, researchers preferred the selected promoter to be limited to the ductal epithelial or exocrine cells. Most single genetically modified models cannot reproduce the whole process of pancreatic tumorigenesis, and the progression from the normal epithelium to cancer cells often requires four to five genetic mutations 19. Additional genetic modifications, such as *P53* and *P16* inactivation, can accelerate tumorigenesis and metastasis. Conditional gene knockout technology allows gene modification to be limited to a certain part or a certain stage of development, so the time and space of the mutant gene can be accurately contolled, enabling more accurate study of gene function.The Cre/loxp recombinase 20 and tet-on systems 21 are the most commonly used conditional gene knockout strategies 22. GEMMs of pancreatic cancer are similar in nature to thehuman disease. In particular, their metastasis pattern is the most similar to that of human pancreatic cancer. The model can be used to study early-stage tumor formation, allowing researchers to ascertain tumor pathogenesis and the effects of therapy. However, the model is limited because it is genetically and biologically different from the human tumor, its modeling time is difficult to control, and its cost is high. Furthermore, it is difficult to meet experimental requirements in terms of quantity.

#### KIC model (Pdx1-Cre, LSL-Kras^G12D^, Ink4a/Arf^lox/lox^)

The Pdx1(pancreatic duodenal homeobox-1) gene which expressed in pre-pancreatic endoderm starting at embryonic stage, would express in acinar and other endocrine cells during development, thus Pdx1-Cre could drive gene modifation in all pancreatic cell types 23. Based on Pdx1-Cre mice, several spontaneous pancreatic cancer models were established. Among those models, KIC is the most notable one. The deficiency of cyclin-dependent kinase inhibitor 2A (*Cdkn2a, Ink4a*) gene, whose inactivtion is associated with melanoma-pancreatic cancer syndrome in human, would not couse the spontaneous pancreatic cancer. But combined with pancreas-specific Cre-mediated activation of a mutant *Kras* allele (*Kras^G12D^*) can result in an earlier appearance of PanIN lesions and these neoplasms progressed rapidly to highly invasive and metastatic cancers (duodenum, stomach and spleen), resulting in death in all cases by 11 weeks 24.

#### KPC model (Pdx1-Cre, LSL-Kras^G12D^, LSL-Trp53^R172H/+^ )

Higorani's team has targeted concomitant endogenous expression of *Trp53^R172H^* and *Kras^G12D^*to the mouse pancreas, revealing the cooperative development of invasive and widely metastatic carcinoma that recapitulates the human disease 25. In such model, the spontaneous cancer in pancreas can cause liver and lung metastasis about 2.5 months. Many of the classical features of malignancy in general and of pancreatic cancer in specific can be recapitulated by *Ink4a/Arf* loss in the setting of *Kras* activa- tion.

#### KD model (Pdx1-Cre, LSL-Kras^G12D^ , Smad4^lox/lox^)

Smad proteins are phosphorylated and activated by transmembrane serine-threonine receptor kinases in response to transforming growth factor (TGF)-beta signaling, and its inactivation is common in pancreatic cancer. Some researchers have targeted oncogenic *Kras* expression and conditional *Smad4/Dpc4* deletion to progenitor cells of the murine pancreas 26, 27. They found that most mouse had IPMN lesions in pancreas with the slow progression of tumor.

#### PDAC model by TGFBR2 knockout with Kras (Prf1a-Cre,LSL-Kras^G12D^ ,Tgfbr2^lox/lox^)

Pancreas associated transcription factor 1a (*Prf1a*) plays a role in determining whether cells allocated to the pancreatic buds continue towards pancreatic organogenesis or revert back to duodenal fates. The protein is thought to be involved in the maintenance of exocrine pancreas-specific gene expression including elastase 1 and amylase. Mutations in this gene cause cerebellar agenesis and loss of expression is seen in ductal type pancreas cancers 28. TGFBR2 is a transmembrane protein that has a protein kinase domain, forms a heterodimeric complex with TGF-beta receptor type-1, and binds TGF-beta.TGF-beta signaling plays an important role in PDAC progression, as indicated by the fact that *Smad4*, which encodes a central signal mediator downstream from TGF-beta, is deleted or mutated in 55% and the type II TGF-beta receptor (*Tgfbr2*) gene is altered in a smaller subset of human PDAC. The *Tgfbr2* knockout combined with *Kras(G12D)* expression developed well-differentiated PDAC with 100% penetrance and a median survival of 59 days 29. And the clinical and histopathological manifestations of the combined *Kras(G12D)* expression and *Tgfbr2* knockout mice recapitulated human PDAC. Such models indicate that blockade of TGF-beta signaling and activated Ras signaling cooperate to promote PDAC progression and is better for human to study the TGF-beta signaling in the development of PDAC.

#### Tetracycline-induced TetO-Cre (Figure 1)

Cre expression can be activated when rtTA or tTA with transcriptional activation functions bind to tetO. Binding of rtTA or tTA to tetO is regulated by tetracycline or its derivative doxycycline (Dox). Specifically, tTA only induces Cre expression when it binds to tetO in the absence of Dox; it does no bind to tetO when Dox is present, so Cre is not expressed in such cases. Concisely, rtTA binds to tetO and induces Cre expression when Dox is present; when Dox is absent, it does not bind to tetO, and Cre is not expressed. Thus, in tetO-Cre and tissue-specific rtTA (or tTA) double-transgenic mice, Cre recombinase can be controlled in space and time by administering or withdrawing Dox. Cre recombinase specifically recognizes the loxp site and cleaves the DNA sequence, causing DNA sequence recombination between the two sites.

## Establishment of animal models based on cell lines

To understand certain aspects of human pancreatic tumors, such as tumor growth, metastasis, drug efficacy, *etc.*, researchers generally prefer the athymic (nude) mouse,which is a mutant mouse said to be nude because it is hairless due to the presence two copies of the gene "nu" (for nude). Nude mice have no thymus and therefore no T cells, a class of lymphocytes that depend on the thymus to develop. For lack of T cells, nude mice cannot reject tumors or transplants of cells from humans or other animals. The phenotype of the original tumor can be maintained after cancer cells of human origin have been implanted into such models, although some abnormal reactions will occur 30. However, one recent study used severe combined immunodeficiency mice (SCID), which has the biological charateristics of T cells and B cells combined deficiency, to receive pancreatic cancer cells of human origin. The results showed that differences in immunodeficiency do not affect the occurence of pancreatic cancer in mice, and that the potential for metastasis is largely determined by the specific cell line 31.

### 1. Cell line selection

The low diagnostic rate of pancreatic cancer is partly due to a lack of specific molecular changes, so it may be useful for researchers to understand their known cell lines (Table 1). Therefore, before beginning studies on pancreatic tumors, researchers should know what the research direction is. This will allow them to select the appropriate cell line and evaluate its clinical background, growth characteristics in both in vitro and in vivo experiments, and the phenotypic characteristics (adhesion, invasion, metastatic ability 32), and genotypic changes, which most often occur in the* KRAS, SMAD4, TP53*, and *P16* genes (Table 2) 33-36.

**Cell geonotypes:** Studies have shown that mutations in these four genes are not associated with the degree of differentiation 37 or biological behavior 38 of pancreatic cancer cells. However, research does indicate that in vivo tumor metastasisis related to alterations in the *p53* gene, suggesting that genotype is related to the phenotype in pancreatic cancer cell lines 39, 40.

**Cell metastasis and invasion:** The biological characteristics of tumor metastasis can be understood through cancer cell metastasis experiments. In the Boyden chamber invasion model, cells migrated from one chamber to another through the artificial basement membrane pores at different chemokine concentrations 41. Other migration experiments include the transwell and scratch assays 42. Stahle et al. found that PANC-1 cells were five times more active than BxPC-3 cells in the transwell migration experiment 43. Lin et al.evaluated mobility by measuring the phagocytic trajectory of cell movement on a colloid surface; they found that both HPAF-II and BxPC-3 cells had good mobility 44.

**Tumorigenicity:** In a study by Schmidt, a pancreatic cancer cell suspension was injected into nude mice. The researchers then observed the volume, quantity, and metastasis of the subsequent tumor to roughly ascertain the tumorgenicity of the cell line. Relatedly, different methods of tumor induction can cause differences in the tumor formation rate and metastatic colonization location. For example, intra-abdominal or intravenous injection, *in situ* implantation, and implantation metastasis show differing outcomes. Subcutaneous injection of tumor cells is the most common experimental method, probably because it is easy to operate. Different cell lines result in tumors of significantly different sizes. In one study, Capan-1, PANC-1, and MIA PaCa-2 cell suspensions were injected into the severe combined immunodeficiency (SCID) mice. After 30 days, a biopsy was taken, revealing the tumor sizes in the following oder: MIA PaCa-2 > Capan -1 >PANC-1^45^. Eibl *et al*. 46 uesd donor nude mice to grow Capan-2 and MIA PaCa-2 tumors. They then removed the tumor, cut it into a cube of 1×1×1 mm^3^, and implanted it in the pancreatic tail of recipient nude mice. They reported a 100% tumor formation rate and that MIA PaCa-2 tumors grew faster. However, because the tumor was first formed under the skin, this in situ tumor implantation model lacks the changes related to the tumor microenvironment and morphology of early-stage tumor. Direct injection of cancer cells into the pancreas can better reflect the tumorigenesis and development of pancreatic cancer. Indeed, several studies have focused on direct injection of different pancreatic cancer cell lines into the pancreas of SCID mice to induce tumor formation 32. The tumor gomation rate were as follows: AsPC-1, 100% (10/10); CFPAC-1, 100% (10/10); HPAF- II, 100% (8/8); Capan-2, 90% (9/10); Hs 766T, 90% (9/10); HPAC, 88% (7/8);PANC-1, 80% (8/10); and BxPC-3, 67% (6/9).

### 2. Establishment of a transplanted tumor model

#### 2.1 Orthotopicimplantation models

***In situ* tumor formation:*** In situ* pancreatic cancer can be induced using in situ injection or pancreatic capsule implantation of tumor cells. In the latter case, tumor cells grow subcutaneously for 4 weeks to form a tumor. The tumors are then excised and cut into pieces of 1~2 mm^3^. In recipient mice, the pancreatic capsule is then opened, and the tumor is implanted into the tail of the pancreas. The tumor formation period is 4 weeks, and the rate is 100%; the injection of tumor cell suspension has a lower tumor formation rate than the transplantation method, and the injection port is likely to cause cell shedding, resulting in extensive transplantation metastasis. For this reason, the method is rarely used 47. However, researchers have implanted pancreatic cancer cells into a recently developed thermosensitive biogel. The cells then develop into tumors. The gel is liquid at a low temperature and turns into jelly at body temperature, which prevents cell shedding; the gel can also dissolve any intervention drugs and is an excellent model for studying such drug. In general, *in situ* tumor formation of pancreatic cancer can fully simulate the internal environment of tumorigenesis and development, and it can affect the whole body during the tumor evaluation period.With the *in situ* tumor model, the tumorigenesis time is short and the tumorigenesis rate is high, so the original tumor structure is maintained, as are most biological characteristics of the human tumor, including the growth of primary tumor, local invasion, and subsequent distant visceral metastasis. The model is an indispensable for studying the tumor microenvironment and is important for exploring new surgical approaches, nutritional support, and other ancillary treatments for pancreatic cancer.

#### 2.2 Ectopic implantation of pancreatic cancer

The classical simplification of metastasis into an orderly sequence of basic steps—local invasion, intravasation, survival in the circulation, extravasation and colonization—has helped to rationalize the complex set of biological properties that must be aquired for a particular malignancy to progress towards overt metastatic disease. These biological events have been described 48, and many genetic and epigenetic events have been identified that contribute to the metastatic path. In all of the metastatic models, pancreatic cancer cells can survive in the circulation, such as lymphatic or blood vascular channels, then lodge in capillaries at destination and attach to and through endothelium. Finally, tumor cells can proliferate and grow as masses. However, in the implantation tumor models, there are still several models that can simulate the whole process of tumor metastasis, such as injection of cells orthotopically into the pancreas and metastasis to liver or lung, as well as spleen injection and metastasis to liver indirectly.

**Subcutaneous tumor formation:** The most common ectopic site of injection is the subcutis. The primary reason for this is convenience: subcutaneous injections are easy to perform, and tumors are readily visible for monitoring growth. This model involves planting tumor cells or tumor tissue directly under the skin of mice. Nude or other immunodeficient mice are generally used in such experiments to study the biological behavior of tumors and intervention therapy. The model is easy to operate, inflicts little trauma on the mice, and confers a high tumor formation rate (80%-100%). The implantation sites are usually located in the back, neck, armpits, groin, or other areas with a rich supply of blood and lymphatic vessels. The model uses tumor cells in the logarithmic growth phase. Briefly, the cell suspension density is adjusted to 1-2×10 ^7^/mL using PBS, and the cell suspention is injected into the implantation site at a volume of 0.2 mL. The mice are then fed in cages. The tumor formation rate and size differ depending on the cell line used. Although subcutaneous tumor formation is easy to operate and suitable for large-scale experiments, it is limited to subcutaneous growth, without distant metastasis, or internal organ invasion, and it cannot truly reflect the tumor microenvironment of pancreatic cancer. In this way, the model does not match the real human pancreatic cancer, and it is therefore used to assess the response of tumors to specific drugs, including antibody-based and cellular drugs, but not for mechanism studies.

**Liver metastasis model:** At the time of presentation, patients withpancreatic cancer are usually at an advanced stage, with tumor invasion into adjacent structures or metastasisinto the peritoneum via direct extension, as well as into the regional lymph nodes or distant organs, such as the liver and lungs 49. The most commonly used liver metastasis models involve spleen injection and direct intrahepatic implantation. In such models, the spleen is injected with a pancreatic cancer cell line at the logarithmic phase, and a 1×10^6^/mL single-cell suspension is prepared using ice-cold sterile PBS. Experimental animals are then anesthetized and disinfected, and the spleen is exposed at a distance of 0.5 cm left of the ventral midline. Next, 100 μL of cell suspension is injected slowly using an insulin syringe. Immediately after injection, tissue glue or an alcohol cotton ball are used to prevent bleeding and transplantation metastasis into the abdominal cavity. This liver metastasis model is mainly used to study the invasive ability of pancreatic cancer; it is not applicable to the study of blood flow dissemination. The intrahepatic implantation model is a supplement to the model. In this model, the tumor cell suspension is directly injected into the liver through the portal vein. Tumor tissue from human or experimental animals can then be cut into a 1-mm^3^ tumor mass and directly implanted under capsule of the left lobe using a 16-gauge needle. The above models can complement each other and be used to systematically study various cascade processes in which pancreatic cancer develops from the primary tumor, invades and migrates intothe blood vessels, and acclimates the microenvironment of the metastatic tumor, allowing the secondary tumor to grow.

**Lung metastasis model:** The lung metastasis model is established by injection of tumor cellsthrough the tail vein. After the tumor cells enter the capillary network of the lungs through the systemic circulation, they gather in the microvessels of the lungs, and metastatic tumors 1~2 mm in diameter are formed in the lungs after around 1 month. By labeling tumor cells with fluorescent proteins, tumor colonization and growth can be continuously observed under an in vivo imaging system. This method also causes tumor formation in ograns other than the lungs, such as the liver, so this method is also used to study the hematogenous metastasis.

**Lymph node metastasis model**: The presence or absence of lymphatic metastasis has a guiding role in the treatment of pancreatic cancer, but no imaging method or technique can satisfactorily track lymph node metastasis 50, 51. Therefore, to better study this phenomenon, a stable lymph node metastasis model for pancreatic cancer is needed. No cell lines have been reported to confer specific lymph node metastasis, and researchers usually screen for such cell lines by continuous screening and planting *in vivo*. For example, Li et al. used the BxPC-3 cell line to produce a highly lymphatic metastatic pancreatic cancer cell line, dubbed BxPC‐3‐LN5, through repeated screening. They then injected 100 µL of 1×10^9^/mL cell suspension into the left hindpaw of BALB/C nude mice and observed swollen lymph nodes in the popliteal fossa of the left knee after about 5 weeks 52.

**Perineuronal invasion model:** Patients with pancreatic cancer often have severe pain due to peripheral nerve invasion, which considerable impacts quality of life. Pancreatic cancer has a high incidence of invasion and metastasis into the nerves and plexuses surrounding the arteries, and this is one e important factors in local recurrence of pancreatic cancer after excision. Therefore, reseachers must further explore perineuronal invasion of pancreatic cancer, with a view to reduce patient suffering and improve clinical treatment. Both human and mouse perineuronal invasion models of pancreatic cancer are used. In the former case, the celiac plexus and superior mesenteric artery nerve are obtained from a donor 6 hours after death by postmortem autopsy. Under aseptic conditions, the nerves are then cut into1-cm pieces and immediately placed in RPMI-1640 medium containing antibiotics. The isolated tissues are implanted subcutaneously in non-obese diabetic (NOD)/SCID mice. After 4 weeks, 7×10^6^ pancreatic cancer cells are injected near the plantation site. After 5 to 8 weeks, the tumor volume is around 1.5 cm^3^. The mouse model also uses NOD/SCID mice: 7 × 10^6^pancreatic cancer cells are injected into the midline of the mouse. In this model, it is better to choose a cell line with a tendency towards perineuronal invasion, such as Capan-1 or Capan-2 53, 54.

## Patient Derived Tumor Xenografts (PDTX)

Xenograft models are either created from injecting patient-derived cell lines into immunocompromised mice or from implanting a fragment of the tumor (PDX) into these animals. In the latter model, researchers implant small tumors from a patient's pancreas into experimental immune-compromised mice, simulating their native growth environment 55, 56. Tumors cultured using this method can better preserve matrix heterogeneity and retain more human tumor matrix components in the early generations (within 10 generations) 57. They can also retain the histological characteristics of the original tumor, such as morphology, lymphatic and vascular systems and necrotic areas 58. Moreover, they retain molecular diversity, with at least the first 10 generations showing microarray-comparative genomic hybridization, microsatellite instability, and higher genetic stability—gene sequencing shows that neither the DNA copy number nor the gene expression profile differs significantly between the early and late generation models 59. This model can reflect the tumor characteristics in individual patients and is necessary to study individualized treatment. However, the cycle time is long and the model's success rate is low. In addition, the most typical feature of pancreatic cancer is rich stromal cells. With the passage of the tumor, the human stromal cells in the tumor are gradually replaced by the mouse cells, so they still cannot truly reflect the original biological behavior.

## Establishment and application of pancreatic cancer organoid

Cell lines, genetically engineered mouse models and transplanted tumor models all have important clinical significance and scientific research value, but each also has clear shortcomings, especially with regards to individualized treatment. The establishment of xenograft tumors requires effort and time, as well as materials. In addition, in situ tumor models based on cell linesnever truly reflect the patient's condition. Organoid models are artificially control lable and can reproduce the three-dimensional structure of PDAC;it has attracted increasing attention because it can overcome the limitations of the traditional model. Organoids can be used to study tumorigenesis and tumor development, including the solid and interstitial components of the tumor, and also as a "test bed" to help determine specific treatment options for patients using *in vitro* testing.

*In vitro* culture of the pancreas can be traced back to 1938, when Carrel and Lindberg used the irrigation method to culture a cat's pancreas *in vitro* for 4 weeks 60. In the 1980s, researchers began to explore how to culture isolated pancreatic cells in a three-dimensional structure 61. On the basis of previous experience, Speier *et al.* sliced ​​the pancreas of the mouse and then successfully cultured it for 7 days in agarose 62; the normal human pancreas and pancreatic tumors can be cultured in the same way for 6 days 63. In a further improvement of this method, part of the normal pancreas and tumor were placed in a collagen or matrix gel and used for drug sensitivity testing 64. In addition, PDAC cell lines have been directly cultured in a three-dimensional structure 65, using various physical methods to prevent cell adhesion and form a polarized spheroid structure. Lorenzo Moroni's teamwere aimed to investigate the interactions between human primary PDAC cells and take polymeric scaffolds with different design and composition to create biomimetic models of PDAC 66. The cultivation of pancreatic cells in a three-dimensional space has allowed researchers to realize the possibility of organoids, but no uniform definition of organoids has yet been agreed.

Clevers* et al.,* working with Tuveson Laboratories 67, found that cells isolated from PDA or PanIN lesions in mice can be cultured into organoids. They prepared pancreatic ductal organoids from multiple murine primary tumors (mT) and metastases (mM). Orthotopic transplantation of mT organoids initially generated low- and high-grade lesions that resembled mPanINs. Over longer periods of time (1-6 months), transplants developed into invasive primary and metastatic mPDA. Similarly, this kind of tumor model is applicable to human pancreatic cancer cells. They researchers modified the culture conditions to support human normal and malignant pancreatic tissues. These patient-derived organoids (PDO) can be cryopreserved and passaged indefinitely, and they can be genetically, transcribed, proteinized, and biochemically analyzed. Therefore, this system is an ideal model for exploring tumor progression at each stage. Melissa Skala *et al.* 68 used a similar method to isolate PDA cells in transgenetic mice with the following genotype: *Ptf1a Cre/+; Kras LSL-G12D/+, Tgfbr2 fl/fl* mice. These cells were cultured in mixed medium and serum-containing medium to develop into an organoid. This method can be used to culture tumors that have been removed from human pancreatic cancer.

Senthil Muthuswamy *et al.* 69 established three-dimensional culture conditions to induce differentiation of human pluripotent stem cells into exocrine progenitor cells, forming ductal and acinar structures *in vitro* and *in vivo*; they also identified culture conditions for cloning freshly collected PDAC cells into tumor organoids, which can maintain the differentiation status, histological structure, and phenotypic heterogeneity of the primary tumor, as well as preserve the unique physiological changes seen in the patient, including hypoxia, oxygen consumption,epigenetic marks, and sensitivity difference to histone methyltransferase EZH2 inhibition.

Calvin Kuo *et al.* 70 used an "air-liquid interface" (ALI) method in which embryonic tissue fragments were cultured in type I collagen gels built on a permeable substrate with a medium underneath that allows nutrients to diffuse from the bottom. The top of the medium was exposed to the air so that the cells could obtain a higher level of oxygen than in conventional culture methods, thereby preventing hypoxia. In the ALI culture, a pancreatic tissue from newborn mice formed an organoid surrounded by stromal cells and containing ductal epithelial cells. It could survive for 50 days without exogenous growth factors, but cannot be passaged. Later, the researcherscultured pancreatic organoids from* Kras^LSL-G12D/+^*and *Trp53^fl/fl^* mice.

In most organoid studies in the cancer field, primary carcinoma samples have been generated under adult stem cell (ASC)-organoid conditions. However, CRISPR mutagenesis technology has been applied to pluripotent stem cell (PSC)-based organoids to generate cancer-causing mutations. Organoid cultures allow several parameters to be observated: (1) interpatient variation can be captured and maintained, (2) patient material can be xenotransplanted with high efficiency, (3) the drug response of the corresponding patient can be faithfully reproduced, and (4) drug sensitivities of PDOs can be recapitulated in PDX settings. The organoid model is highly efficient, so a corresponding organoid biobanks can be established on the basis of different tumor types. Indeed, several studies have reported that organoids can be derivedfrom needle biopsies taken from liver cancer 71, pancreatic cancer 72, 73, or humancolorectal cancer metastases 74. In the studies of colorectal cancer, two laboratories separately have established human intestinal cell organoids containing mutant tumor suppressor genes and oncogenes, which can be used to study the mechanism of tumorigenesis and invasion 75, 76. In the near future, pancreatic organoids will likely play a key role in the development of precision medical treatment against PDAC, which will have its own unique advantages.

## Discussion

Many studies revealed that pancreas-specific *Kras* mutantion can induce spontaneous pancreatic cancer in experimental animals. Meanwhile, the mutantions of *Kras* are found in more than 90% of human pancreatic ductal carcinoma specimens 77. The most frequent mutation is the constitutively active *KRAS^G12D^* allele 78. Thus the study based on *Kras* mutated models can facilitate researchers to understand the tumorigenesis and development of pancreatic cancer. However, animal models based on single mutation of *Kras* might be unsufficient to explore the landscape of pancreatic cancer biological behivorson account of its tumorigenesis and development is an extremely complex and long-term process. Epidemiological data also indicates that smoking, high-calorie diet, chronic pancreatitis, and type 2 diabetes can increase the risk of pancreatic cancer, which prompts that the oncogenesis and progression of pancreatic cancer is a multifactorial process. This proess is a result of the interaction of oncogenes, tumor suppressor genes, metabolic environment, immune system, *etc*. However, until now there is no effective and reliable animal models can completely simulate the pathological process of pancreatic cancer. At present, a practical and feasible method is to combine several diffirent models, such as pancreatic cancer model with type 2 diabetes induced by injection of STZ into KIC mice with high-fat diet, panreatitis-pancreatic cancer model established by bombesin injecting into KIC mice, and KIC mice intervened with smoking, high-fat or high-chelesterol diet. All of these models aim to explore the key biology event of pancreatic cancer. In addition, iDTR-CRE system in the pancreatic cancer model can achieve the depletion of a certain immune cell subsets. By this system we can explore which immune subsets play a pivotal role in maintaining immune surveillance and anti-tumor function in the process of pancreatic cancer. Furthermorewith the development of *in vivo* screening CRISPR/Cas9 technology genes' noval function may be identified directly in the pathogenesis of pancreatic cancer, including non-coding RNAs. And new pancreatic cancer related animal model may be established. Now main current view summarizes the occurrence of pancreatic cancer as a consecutive biological event: *Kras* mutation and *Her2* overexpression could cause intraepithelial neoplasia of the pancreas, tumor suppressor genes *p16, p53, DPC4* and *BRCA2* may dysfunction in the immunol suppressive microenviroment, eventually leading to pancreatic cancer. Many effective therapies have been developed for pancreatic cancer burden mice, which can control pancreatic cancer in animal models and even eliminate tumors. However, the current clinical challenge is that pancreatic cancer is still difficult for early detection, lack of effective treatment and very poor prognosis. Pancreatic Ductal Adenocarcinoma's five-year survival rate is still less than 10%, the majority of patients already lost surgery opportunity when found. The present dilemma is mainly reflected in two aspects: firstly, no effective biomarkers of pancreatic cancer have been found in the current study. How to use animal models combined with circulating tumor cell monitoring technology, cfDNA sequencing technology, metabolomics and so on to find new tumor markers is the future research should be concerned. Secondly, the study of single gene mutation animal models will inevitably lose sight of one another, and it will be difficult to exert the landscape of tumor. Only by introducing multiple pathogenic factors into animal models and leading multi-target therapy strategies, especially introducing microsurgery intervention, all of these above will be possible to provide practical theoretical basis for the clinical treatment of pancreatic cancer.

It is true that tumor associated microenvironment plays an important role in the development and progression of cancer. Researchers have recognized that every process of tumor is driven by cooperation between cancer cells and their microenviroment, including relevant fibroblasts, immune cells and other specific interstitial cells. Because of the specificity of pancreas, pancreatic cancer microenviroment includes numerous fibroblasts, pancreatic stellate cells, nerve tissue, immune cells and vascular related cells. These different types of cells not only provide survival soil for the proliferation and malignant evolution of cancer cells, but also are important factors for pancreatic cancer to escape immune surveillance and even "counteract" the immune system. How to realize "mimics" or even "humanizated" of pancreatic cancer microenvironment in animal models is a key scientific issue worthy of attention. Although it is difficult to humanize the tumor microenvironment in animal models, the maturity of several new technologies makes this assumption possible. For example, the immune system of patients can be individually reconstructed in severe immunodeficient mice such as NCG/NSG through the method that small molecule compound cocktail inducing long-term expansion of hematopoietic stem progenitor cells *in vitro* 79, which can also help to the explore the individualized mechanism of tumor immune escape in PDX model.

## Conclusion

Because pancreatic cancer shows no specific early clinical manifestations and has high mortality, medical researchers find it difficult to study the biological behavior and internal mechanisms of early pancreatic cancer, and our understanding of the mechanism underlying tumorigenesis is limited. Early diagnosis allows patients to receive timely treatment in the curable phase. Use of experimental animal models is an important method for gaining insight into the etiology, risk factors, prevention, and treatment of this tumor. Although many mouse models can be obtained using transgenic technology, there is still a lack of specificity for clinical research.

Perhaps importantly, 70% of pancreatic cancers are induced by carcinogens, with nitrosamine and polycyclic aromatic hydrocarbons in tobacco being high risk factors for inducing pancreatic cancer. Therefore, to induce tumorigenesis of pancreatic cancer, chemically induced models are more useful. However, the transplantation tumor model has been used to study etiology, diet, modification factors, and some natural products, as well as early diagnosis, prevention and treatment of pancreatic cancer.

In summary, current animal models can mimic the characteristics of most human pancreatic cancers, but no model has become a “gold standard” that meets the needs of all research. By simply focusing on specific needs and combining the characteristics of each model, researchers can better study the overall process of tumorigenesis and development of pancreatic cancer. Ultimately, to reduce PDAC mortality, judgments based on genetic and non-genetic risk factors must be improved. As such, researchers must explore new biomarkers and high-resolution imaging techniques to screen for patients with early-stage, high risk cancer, and must carry out drug interventions to prevent PDAC progression and prolonging survival time. In the past few decades, improvements in animal models have driven advances in these areas, and these models will continue to make significant contributions in the coming years.

## Figures and Tables

**Figure 1:**
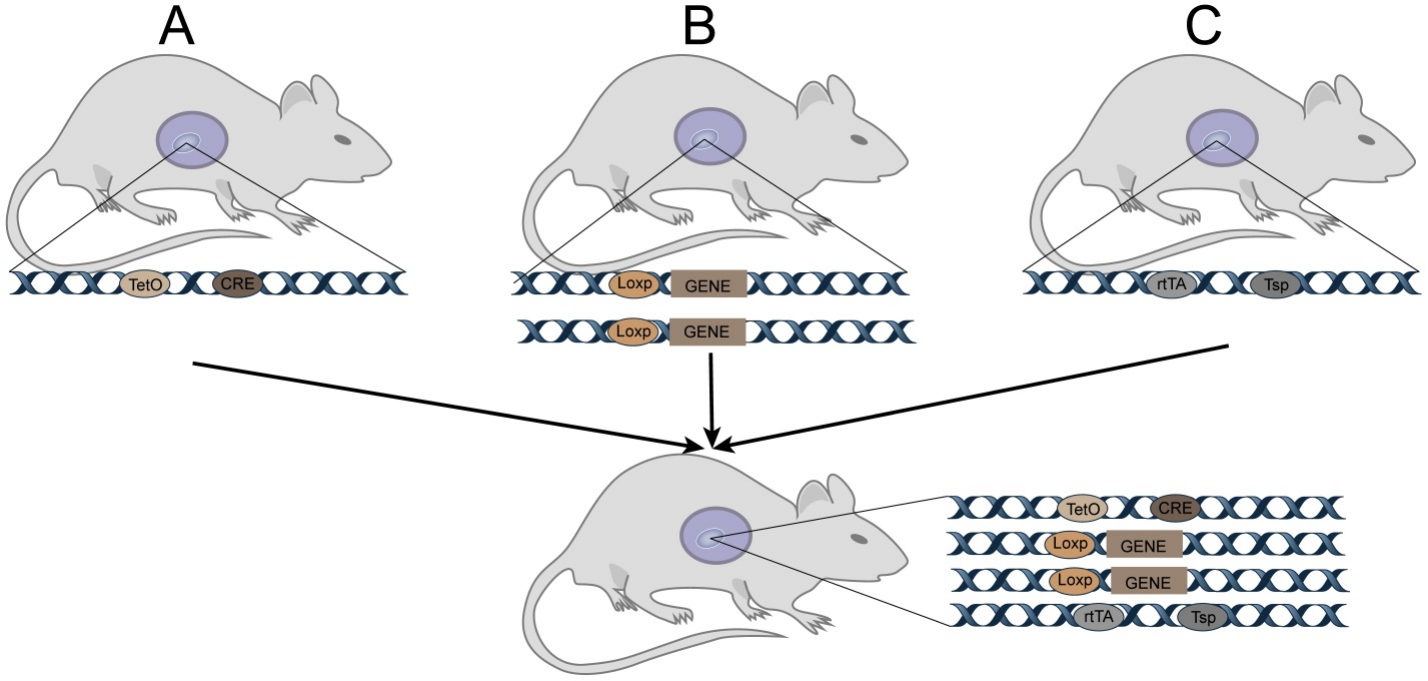
Tetracycline-induced TetO-Cre for GEMM. A: Cre mice (TRE-Cre, also called tetO-Cre) controlled by a tetracycline-responsive element (TRE, also called tetO). C: Mice expressing a tetracycline-responsive transcriptional activator rtTA or tTA driven by a tissue-specific promoter.

**Table 1:** Human pancreatic cancer cell lines

Cell line	Tissue origin	Metastasis	Doubling time	Differentiation degree	Morphology	Tumor formation rate (subcutaneous)	Ref
**AsPC-1**	Ascites	Yes	38-40hrs	Poor	Epithelioid		80
**HPAF-II**	Ascites	Yes	42 hrs	Moderate	Epithelioid		81
**HPAC-1**	Primary tumor	-	41hrs	Good	Epithelioid		82
**MIA PaCa-2**	Primary tumor	-	40hrs	Poor	Epithelioid	66%	83
**PANC-1**	Primary tumor	Yes	52hrs	Poor	Epithelioid	86%	84
**BxPC-3**	Primary tumor	No	48-60hrs	Moderate-Poor	Epithelioid	100%	85
**Capan-2**	Primary tumor	No	96hrs	Good			86
**Capan-1**	Liver Metastasis	Yes	-	Good	Epithelioid		87, 88
**SU.86.86**	Liver Metastasis	Yes	77hrs	Moderate-Poor	Epithelioid		89
**CFPAC-1**	Liver Metastasis	Yes	31hrs	Good		100%	90
**Suit-2**	Liver Metastasis	Yes	29-38 hrs				91, 92
**SW1990**	Splenic Metastasis	Yes	64hrs			100%	93
**Hs766T**	Lymphatic Metastasis	Yes	6-7days	-	Epithelioid		94
**Colo357**	Lymphatic Metastasis	Yes	21 hrs	Good			95
**T3M4**	Lymphatic Metastasis	Yes		Moderate			96

**Table 2:** Animal-origin pancreatic cancer cell lines

Cell line	Organism	Carcinogen	Differentiation degree	Gene mutation	Ref
**PC1**	mouse	BOP	Good	K-ras, P53	97, 98
**WDPaCa**	mouse	BOP	Good	P53	99
**PDPaCa**	mouse	BOP	Poor	k-ras	99
**HPC**	mouse	BOP	Poor		100
**HP1**	mouse	BOP			101
**HaP-T1**	mouse	BOP	Good-Moderate		102
**H2T**	mouse	**BHP**		K-ras, P53	103, 104
**HPD(1-3)NR**	mouse	BHP	Moderate	K-ras, P53	102, 105
Pan02	mouse	MCA		K-ras,smad4	106
6606PDA	mouse		Good		107-109
AR42J	rat				110, 111

Note: BHP: N-nitrosobis(2-hydroxypropyl)amine; animal-origin pancreatic cancer cell lines are commonly used in inbred mice of the same origin for allogeneic transplantation. This model is used more frequently in tumor immunology studies and to evaluate single-agent or combination immunotherapy studies.

**Table 3:** Expression of mutant genes in cell lines

Gene	Expression of Cell Line
**KRAS**	Occurred in almost all of the primary tumors of pancreatic cancer, but the BxPC-3 cell line is WT
**SMD4/DPC4**	Capan-2, MIA PaCa-2, PANC-1, SU.86.86 without SMD4 gene inactivation
**TP53**	Its mutation occurs in 50% of pancreatic malignant tumors and is associated with late tumor progression
**CDKN2A/P16**	Basically all pancreatic cancer cell lines have inactivation of the P16 gene
